# Health-related quality of life in people with autosomal dominant polycystic kidney disease: a systematic review

**DOI:** 10.1093/ckj/sfag116

**Published:** 2026-04-23

**Authors:** Matt Gittus, Yanan Zhang, Sue Harnan, Anthea Sutton, James Fotheringham, Albert C M Ong, Olena Mandrik

**Affiliations:** The University of Sheffield, School of Medicine and Population Health, Sheffield, United Kingdom; Sheffield Kidney Institute, Northern General Hospital, Sheffield, United Kingdom; The University of Sheffield, School of Medicine and Population Health, Sheffield, United Kingdom; The University of Sheffield, School of Medicine and Population Health, Sheffield, United Kingdom; The University of Sheffield, School of Medicine and Population Health, Sheffield, United Kingdom; The University of Sheffield, School of Medicine and Population Health, Sheffield, United Kingdom; Sheffield Kidney Institute, Northern General Hospital, Sheffield, United Kingdom; The University of Sheffield, School of Medicine and Population Health, Sheffield, United Kingdom; Sheffield Kidney Institute, Northern General Hospital, Sheffield, United Kingdom; The University of Sheffield, School of Medicine and Population Health, Sheffield, United Kingdom

**Keywords:** ADPKD, autosomal dominant polycystic kidney disease, quality of life

## Abstract

**Background:**

Autosomal dominant polycystic kidney disease (ADPKD) is the most common inherited kidney disorder and a major contributor to kidney failure worldwide. However, the impact of ADPKD on health-related quality of life (HRQoL) across chronic kidney disease (CKD) stages and kidney replacement therapies (KRT) is poorly understood. This study aimed to synthesize existing evidence on HRQoL as measured by patient-reported outcome measures (PROMs) in people with ADPKD, stratified by disease stage and KRT modality.

**Methods:**

A systematic review was conducted using five databases (Medline, Embase, PsycINFO, CINAHL, Web of Science) and Google Scholar to identify studies published between January 2014 and October 2024. Eligible studies reported HRQoL in individuals with ADPKD using generic, kidney-specific, or ADPKD-specific PROMs Study populations were stratified by CKD stage and KRT modality. Scores were adjusted using country-specific population norms matched for age and sex, with population multipliers calculated to express patient-reported outcomes (PROs) as a proportion of the reference population.

**Results:**

Six studies assessed PROs using the Short-Form-36/12 survey. Physical health worsened with CKD progression, corresponding to lower values relative to matched population norms. Mental health showed smaller deviations from population norms. Dialysis patients had the lowest physical health multipliers, while transplant recipients had better physical health it did not improve to early-stage CKD levels. Two studies using the EuroQual 5-Dimension tool had fewer notable differences between CKD stages. Kidney disease and ADPKD-specific scores showed more pronounced declines across CKD stages than generic PROMs, suggesting greater sensitivity to stage-related changes.

**Conclusions:**

This review demonstrates that PROs for individuals with ADPKD are lower in later CKD stages compared with earlier stages, with the largest effect on physical health. Mental health scores were less affected suggesting adaptation over time. Our findings suggest generic PROMs may underestimate the impact of ADPKD compared to disease-specific tools.

## Graphical Abstract

**Graphical Abstract:**
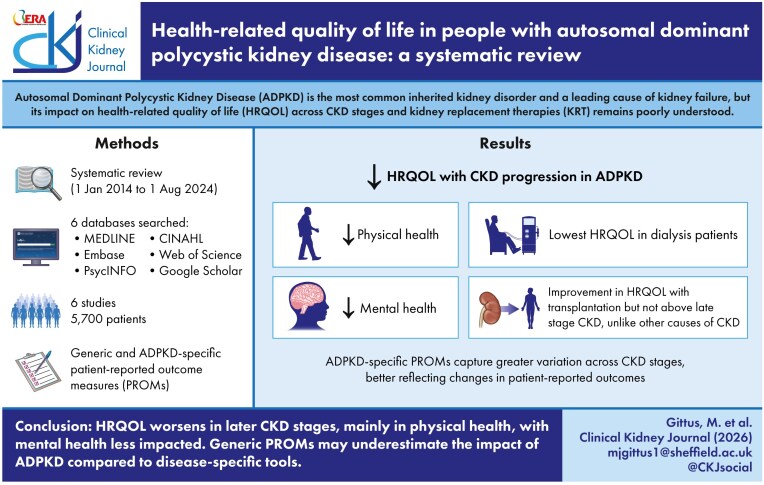

KEY LEARNING POINTS **What was known:**

- Autosomal dominant polycystic kidney disease (ADPKD) is a common inherited kidney disease that affects quality of life through chronic pain, abdominal distension, extra-renal complications, and psychosocial impact.
- Despite the available patient-reported outcome measure tools, no comprehensive synthesis has evaluated how health-related quality of life changes as chronic kidney disease (CKD) progresses in ADPKD.
- This gap hinders understanding of the disease’s full impact on patients over time and limits the ability to tailor interventions to stage-specific quality of life concerns.

**This study adds:**

- Health-related quality of life (HRQoL) decreases with advancing stages of ADPKD, mainly affecting physical health while mental health tends to remain relatively stable.
- Transplantation improves both physical and mental scores but does not restore HRQoL to levels seen in early-stage disease.
- Generic PROMs may not be sufficiently sensitive to capture the impact of ADPKD, which could in turn influence reimbursement decisions in this condition. ADPKD-specific PROMs may better capture symptom burden and disease impact than generic PROMs.

**Potential impact:**

- Findings support the need for a stratified management approach in ADPKD, with interventions tailored to stage-specific burden to preserve physical function and mitigate psychosocial impact over time.
- By highlighting that generic PROMs may underestimate the burden of ADPKD, this study supports the use of disease-specific instruments, which could improve the accuracy of patient outcome assessments and more appropriate reimbursement and health economic decisions.
- Future research should adopt standardized patient classification and employ longitudinal designs to better characterize health-related quality of life changes across CKD stages.

## BACKGROUND

Autosomal dominant polycystic kidney disease (ADPKD) is the most common hereditary kidney disease [1]. It is characterized by the growth of cysts and enlargement of the kidneys, which precede functional kidney deterioration often by several decades [2]. Although symptoms can develop at any age, they typically begin between the ages of 30 and 40 years. These can vary in severity but often include pain, haematuria, abdominal swelling, and extra-renal manifestations such as liver cysts and intracranial aneurysms [3–7]. By the age of 60, around half of all patients with ADPKD develop end-stage kidney failure, requiring kidney replacement therapy (KRT) [2]. Patient-reported outcome measures (PROMs) can provide insights into a patient’s well-being that are unable to be captured by laboratory data alone [8].

Despite the availability of validated PROMs for assessing health-related quality of life (HRQoL) in ADPKD, no comprehensive synthesis has been conducted on how quality of life changes across chronic kidney disease (CKD) stages. Generic PROMs, such as the Short-Form (SF) survey (12 item or 36 item) or EuroQual 5-Dimensions-3 Levels (EQ-5D), enable comparisons across different conditions but may lack sensitivity to the unique manifestations of kidney disease [9]. The SF-12/36 survey records responses as physical component summary (PCS) and mental component summary (MCS) whereas the EQ-5D only reports a single score. Kidney disease-specific PROMs, such as the Kidney Disease Quality of Life (KDQoL) instrument, capture aspects relevant to chronic kidney disease but may not fully reflect the impact of ADPKD-related symptoms. Although ADPKD-specific PROMs, such as ADPKD-Impact Score (ADPKD-IS), ADPKD-Urinary Impact Score (ADPKD-UIS), and Genetic Psychosocial Risk Instrument-ADPKD (GPRI-ADPKD), have been developed to address these gaps, their application and comparative effectiveness across different CKD stages remains unclear. The lack of a consolidated evaluation of patient-reported outcomes (PROs) across CKD stages limits our understanding of the disease’s impact.

This systematic review aims to address this gap by collating and synthesizing existing evidence on PROs at different stages of kidney function for people with ADPKD.

## MATERIALS AND METHODS

### Study design

We conducted a systematic review following the Cochrane rapid review methods guidance [10] and Preferred Reporting Items for Systematic reviews and Meta-analyses (PRISMA) [11]. The protocol was pre-registered on PROSPERO (CRD42024552365). Reporting followed the PRISMA 2020 statement; the checklist is available in Supplementary Table 1.

### Inclusion/exclusion criteria

Studies that included participants classified by CKD stage (stage 1, ≥90; stage 2, 60–89; stage 3, 30–59; stage 4, 15–29; stage 5, <15), non-CKD stage-specific eGFR groupings, dialysis status or transplant status. Studies reporting PROs for people with ADPKD, adults and children, were included. Only peer-reviewed randomized controlled trials and observational studies were included. Case reports, qualitative studies, editorials, protocols, commentaries and conference abstracts were excluded. Systematic reviews were excluded but were screened for additional primary studies.

### Search strategy

Using the Population, Exposure, Comparator and Outcomes (PECO) framework (Appendix 1) [12], ZY developed a search strategy in consultation with a nephrologist (M.G.), systematic reviewer (S.H.), and an information specialist (A.S.) (Appendix 2). Five electronic databases were searched: MEDLINE via Ovid, Embase via Ovid, PsycINFO via Ovid, CINAHL via EBSCO, Web of Science, and the search engine Google Scholar for studies between 1 January 2014 and 1 August 2024. The start date of 2014 was chosen to capture the most contemporary evidence while maintaining a manageable scope for a rapid systematic review. Databases were selected to ensure comprehensive coverage across medical, psychological, nursing, and inter-disciplinary research [13, 14]. Searches were limited to human studies and the English-language due to limited resources for translation, consistent with the scope and capacity of a review conducted by early career researchers. The date of the search was 1 August 2024.

### Study selection

Duplicates were removed in EndNote and results screened in Rayyan. To assess compare screening approaches and ensure consistency between reviewers, Y.Z. and M.G. independently screened a randomly selected 20% sample of studies at the title-abstract stage (*n* = 442). Any disagreements were discussed between reviewers and, if necessary, resolved by a third reviewer (O.M.). The Cohen’s kappa score of 0.95 indicated ‘almost perfect agreement’ between the reviewers [15]. The remaining 80% were screened by a single reviewer (Y.Z.) in line with the Cochrane guidance for rapid review methods [10], which supports partial dual screening when high agreement is demonstrated. Full dual screening was not feasible given time and resource constraints.

### Quality assessment

The Joanna Briggs Institute (JBI) checklist for analytical cross-sectional studies was used for appraisal [16]. One reviewer (Y.Z.) conducted the initial appraisal, which was independently verified by two other authors independently (M.G. and O.M.). The results of the quality assessment were used to inform the interpretation of findings.

### Data extraction

A data extraction form was developed and piloted on two studies prior to formal data extraction. Key study information was extracted by Y.Z. and verified by M.G. and O.M. Data extracted included: general information (title, author, publication year, country); study methodology (study design, study objectives, inclusion criteria, exclusion criteria, PROMs); participant characteristics (sample size, age, gender); and study outcomes (PRO mean scores and standard deviation).

### Data analysis and synthesis

Where multiple studies reported using the same PROM, heterogeneity was assessed using Cochran’s *Q* and the *I*^2^ statistic. High heterogeneity indicated that quantitative meta-analysis was not appropriate; therefore, results were synthesized descriptively without statistical pooling in accordance with the synthesis without meta-analysis (SWiM) in systematic reviews reporting guidance [17]. SF-36 and SF-12 survey responses were described together, as the SF-12 has been shown to reliably reproduce the PCS and MCS derived from the full SF-36 [18]. Where necessary CKD stages within a single study were combined to allow clearer reporting and comparison across studies [19]. For studies where the mean eGFR for a group was not reported the mid-point of the eGFR group was used. For the generic PROMs, population multipliers were calculated by expressing study PRO scores as a proportion of the age and sex-adjusted mean from the corresponding reference population, providing an interpretable measure of disease-related decrement and allowed consistent descriptive comparison across CKD stages and study settings. Standard errors were calculated from available summary statistics to produce 95% confidence intervals. For PROMs where ANOVA was performed, *P* values were adjusted for multiple comparisons using the Benjamini–Hochberg false discovery rate method with adjusted *P* values <.05 considered statistically significant. Not all countries or combinations of countries had reference populations so where this was the case appropriate comparators were selected for illustrative purposes. For SF-36/12, reference populations were from published sources in the UK [20], USA [21], and Japan [22]. For EQ-5D, reference populations were from published sources in Denmark [23], Sweden [24], England [25], and USA[26]. PROMs without sufficient comparators were described narratively. For the PROMs with multiple data collection points, only the baseline PROs were included. All statistical analysis was undertaken in Python v.3 (Python Software Foundation, 2026, version 3.13.2.). Forest plots and line charts were generated using Microsoft Excel (Microsoft 2019, version 16.78.3) to visualize data.

## RESULTS

**Figure 1:**
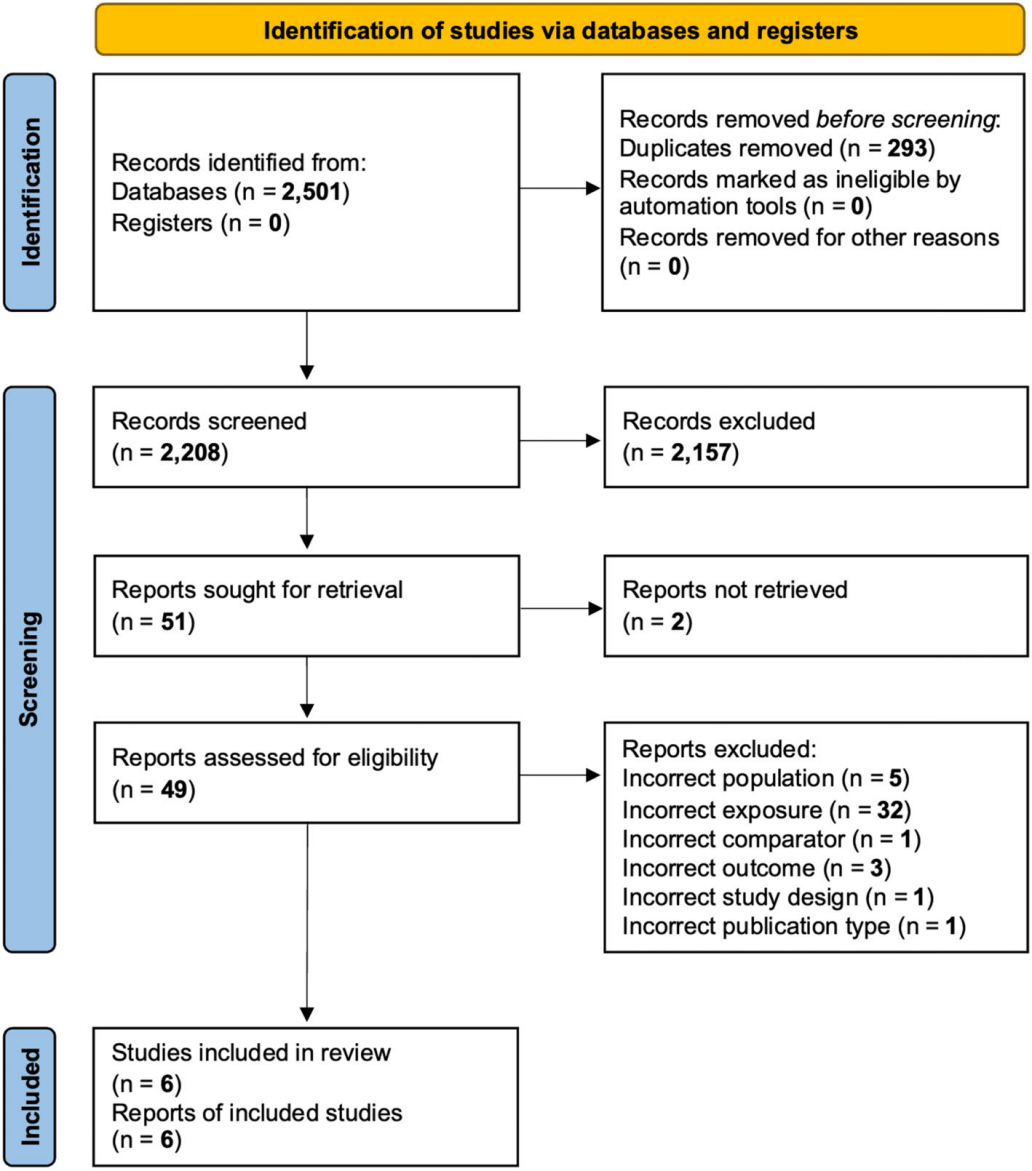
PRISMA flow diagram.

The search identified 2505 records, of which six met the inclusion criteria after screening (Figure 1) [19, 27–31].

### Study characteristics

Study characteristics are summarized in Table 1 and Appendix 3. All studies included were retrospective observational studies with the number of participants per study ranged from 188 in a single centre study [30] to 3409 in a multi-country study [28]. All studies included participants with a diagnosis of ADPKD but only four reported the criteria used for this diagnosis. Participants in the included studies were distributed across the full spectrum of CKD stages and KRT modalities. A combination of PROMs were reported with SF-36 [27, 29–31], SF-12 [19, 28], and EQ-5D [19, 28] used the most frequently (Table 2). These PROMs are described in Supplementary Table 2.

**Table 1:** Summary of the studies included in the review.

						Population characteristics				
Study author (year) [ref.]	Study type	First author country of origin	Countries included (*n*)	Year	Participants (diagnostic criteria)	Sample size	Mean age (SD)	Female sex (%)	CKD stages	eGFR groupings
Eriksson *et al*. (2017) [19]	Observational study	Sweden	4	2017	ADPKD patients (unclear diagnosis criteria)	243	58.0 (12.1)	53.9	1–5, D, T	30-≥90, 15–30, D, T
Miskulin *et al*. (2014) [31]	Observational study	USA	1	2014	ADPKD (unclear diagnostic criteria)	1 043	41.7 (10.3)	49.9	1–4	>60, 45–60, 20–44
Perrone *et al*. (2023) [28]	Observational study	USA	20	2024	ADPKD patients. (imaging criteria)	3 409	45.1 (12.9)	55.5	1–5	≥90, 60–89, 45–59, 30–44,15–29
Simms *et al*. (2016) [27]	Observational study	UK	1	2016	ADPKD patients. (imaging criteria)	349	53.4 (15.8)	58.3	1–5	>60, 30–60, <30
Suwabe *et al*. (2017) [30]	Observational study	Japan	1	2013	ADPKD patients. (imaging criteria)	188	56.7 (9.1)	51.1	T	NR
Winterbottom *et al*. (2022) [29]	Observational study	UK	6	2022	ADPKD patients. (imaging criteria)	465	43.2 (12.8)	55.1	1–3	>90, 60–90, 30–60

D, dialysis; T, transplantation.

**Table 2:** PROMs.

		PROMs						
		Generic			Kidney-specific	ADPKD-specific		
Study author (year) [ref.]	PROMs (*n*)	EQ-5D	SF-12	SF-36	KDQoL-SF1.3	ADPKD-IS	ADPKD-UIS	GPRI-ADPKD
Eriksson *et al*. (2017) [19]	2	✓	✓					
Miskulin *et al*. (2014) [31]	1			✓				
Perrone *et al*. (2023) [28]	4	✓	✓			✓	✓	
Simms *et al*. (2016) [27]	2			(✓)	✓			✓
Suwabe *et al*. (2017) [30]	1			✓				
Winterbottom *et al*. (2022) [29]	1			(✓)	✓			

Brackets indicates that the SF-36 score was collected as part of a combined score KDQoL-SF version 1.3

KDQoL-SFv1.3, Kidney Disease Quality of Life Short-Form version 1.3; SF-12. Short-Form 12-item; SF-36, Short-Form 36-item.

### Quality assessment

Table 3 summarizes the quality assessment. Studies had exposures included, i.e. presence of pain, and all studies clearly defined inclusion criteria and provided detailed descriptions of the study settings, timeframes, and populations. One study included an exposure that was measured in a valid and reliable manner; the other studies did not include an exposure in their study design [30]. Potential strategies to address confounding factors were not reported by three studies [19, 28–30]. Justification for the assessment scores is available in Supplementary Table 3.

**Table 3:** Quality assessment of included studies using the JBI Critical Appraisal Tool.

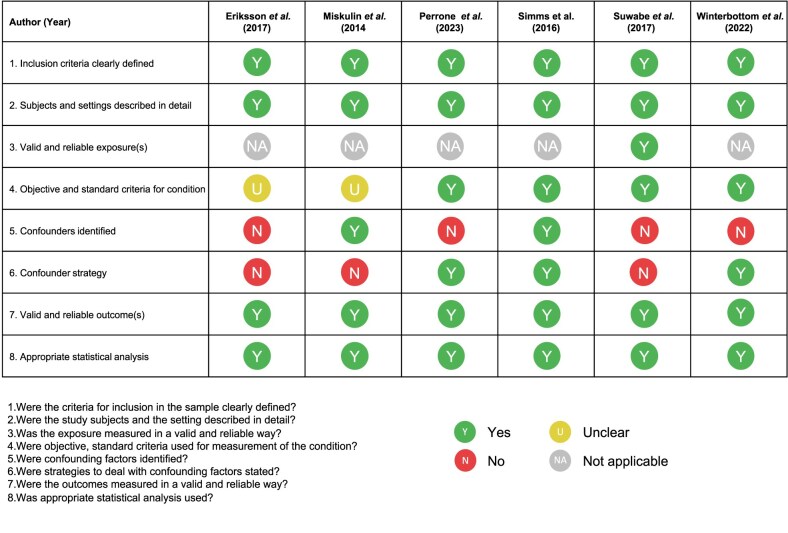

### Chronic kidney disease stage, kidney replacement therapy modality, and patient-reported outcomes

#### Generic PROMs

Assessment of heterogeneity using Cochran’s *Q* and *I*² indicated substantial variation across studies, with an overall *I*² of 99.6% for PCS scores, 99.2% for MCS scores, and 95.8% for EQ-5D scores. This supported our decision to synthesize PRO scores descriptively rather than perform meta-analysis (see Appendix 4 for per-stage estimates).

##### Short-form 36 and 12 (SF-36 and SF-12)

**Figure 2:**
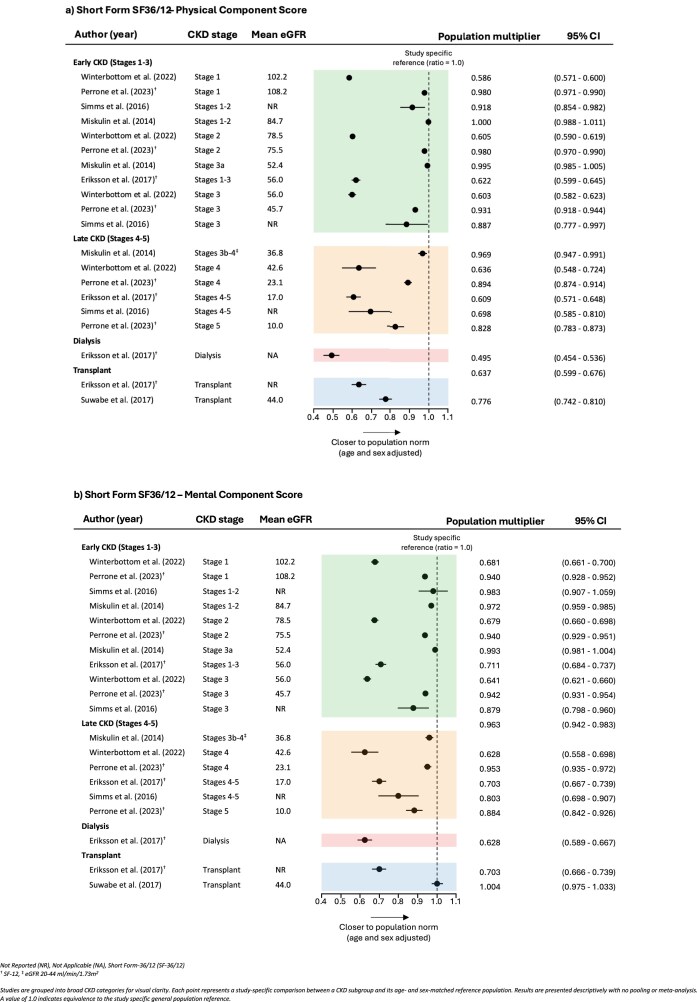
Reported physical and mental component scores (SF-36/SF-12) by CKD stage, dialysis, and transplantation.

All six included studies reported SF survey responses: four using the 36-item survey and two using the 12-item survey. PCS and MCS multipliers differed across CKD stages and KRT modalities (Figure 2). Physical health, generally declined with advancing CKD stage, with population multipliers being lowest among patients receiving dialysis, indicating a substantial reduction in physical health relative to the age- and sex-matched general population. Mental health, as reflected in MCS scores, also varied across CKD stages but showed smaller deviations from the population norm than PCS. Notably, physical health scores in transplant recipients were no better than those reported in late-stage CKD, however higher than for participants on dialysis [19]. By contrast, the mental health score in one study showed an improvement with values that exceeded the population norm [30]. Raw PCS and MCS scores for each study, unadjusted to their reference general population, are presented visually in Appendix 5. Individual SF-12/36 dimensions are illustrated in Appendix 6 and scores for subgroups in each study are available in Supplementary Table 4. Reference populations used to calculate population multipliers are included in Appendix 7.

##### EuroQol 5-dimension (EQ-5D)

**Figure 3:**
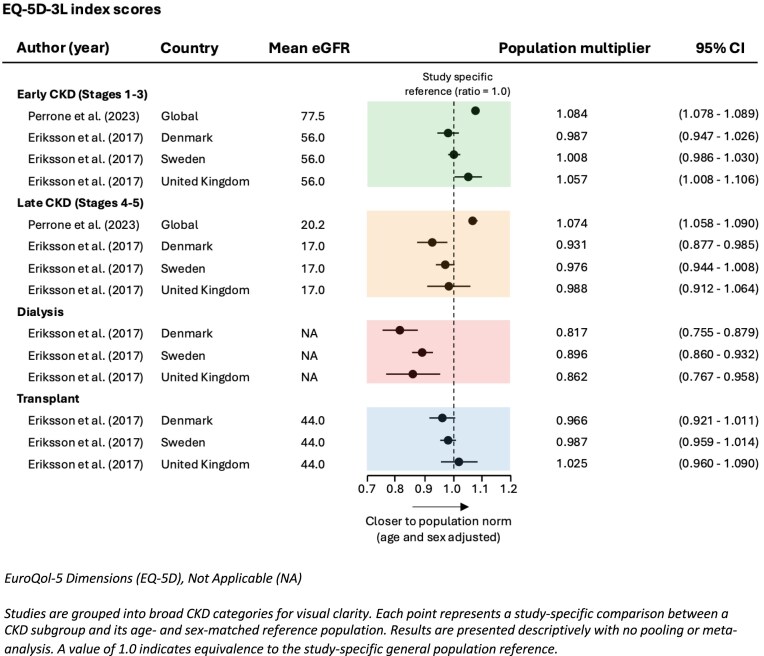
EQ-5D index score by early CKD stage (1–3), late CKD stage (4–5), dialysis and transplantation.

Perrone *et al*. and Eriksson reported declining PROs as CKD progresses using the EQ-5D assessment tool [19, 28], Figure 3. Early-stage CKD participants had the highest EQ-5D scores and dialysis participants (haemodialysis and peritoneal dialysis combined) reported the lowest scores. Utility multipliers above 1 suggest a possible difference in case-mix characteristics between the CKD respondents and the general population, since population utilities are typically lower among older individuals [32], as well as possibly low sensitivity of EQ-5D to reflect the impact of ADPKD on PROs. Transplant recipients had higher population multipliers than dialysis patients but did not reach the levels seen in early-stage CKD, indicating that while transplantation improves physical health but does not fully restore it to the level seen in early CKD. Raw EQ-5D scores for each study, unadjusted to their reference general population, are presented visually in Appendix 5. Reference populations used to calculate population multipliers are included in Appendix 7.

#### Kidney-specific PROMs

**Figure 4:**
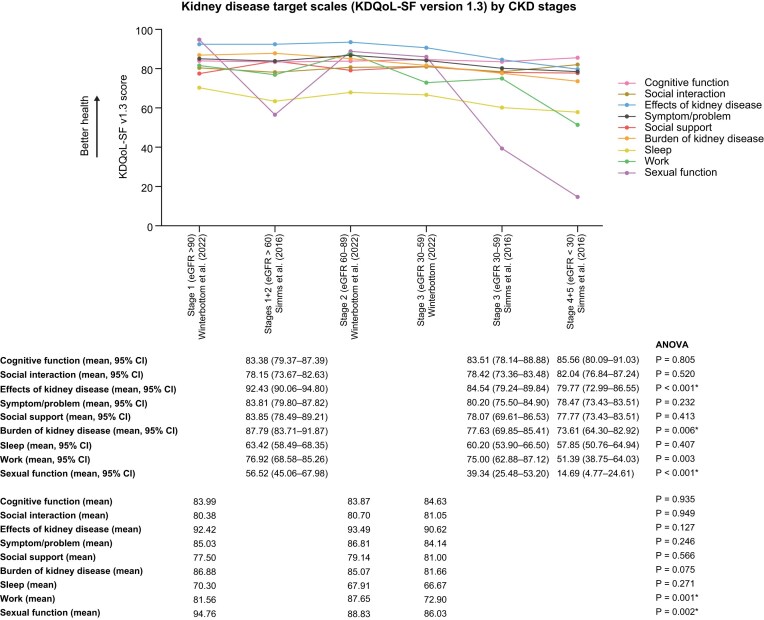
Kidney disease target scales of the KDQoL-SF version 1.3 by CKD stage.

Simms *et al*. and Winterbottom *et al*. assessed PROs in people with ADPKD across CKD stages using the KDQoL-SF 1.3 [27, 29]. This incorporates the SF-36, presented earlier, and kidney disease target scales, presented here. Dimensions of the kidney disease target scales declined with CKD progression, but only sexual function was statistically significant (*P* = .0030, false discovery rate -adjusted *P* = .0267) [27, 29]. Figure 4 illustrates the decline across the dimensions.

#### ADPKD-specific PROMs

**Figure 5:**
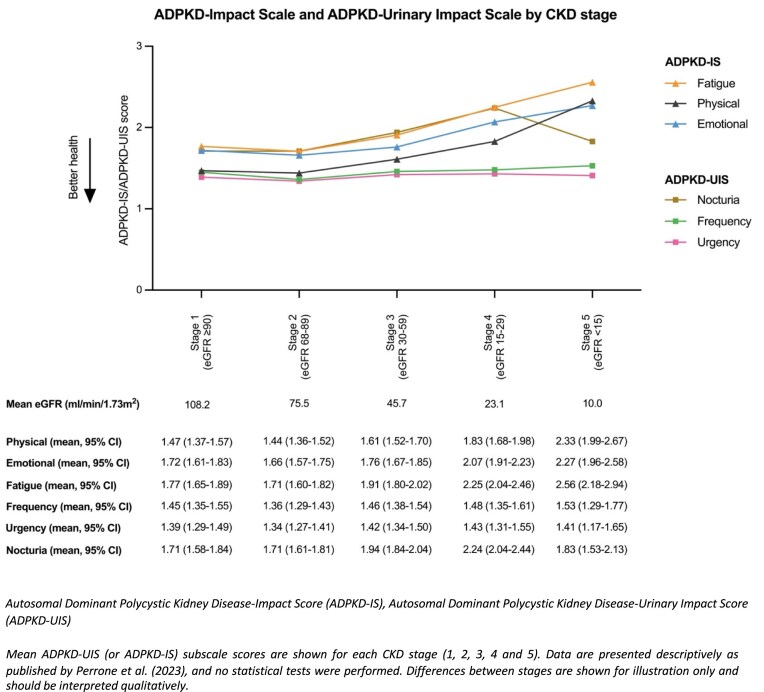
Mean ADPKD-IS and ADPKD-UIS score for CKD stages.

Perrone *et al*. reported both ADPKD-IS and ADPKD-UIS, showing a worsening impact on quality of life as CKD progressed (Figure 5). Total ADPKD-IS and ADPKD-UIS scores worsened from CKD stage 2 onwards, although CKD stage 1 patients had a worse mean score than CKD stage 2. Of note, ADPKD-UIS scores were worse in CKD stage 5 than CKD stage 4. Urinary frequency and urgency scores remained stable across CKD stages, while nocturia worsened up to CKD stage 4 before improving at CKD stage 5 [28].

**Figure 6:**
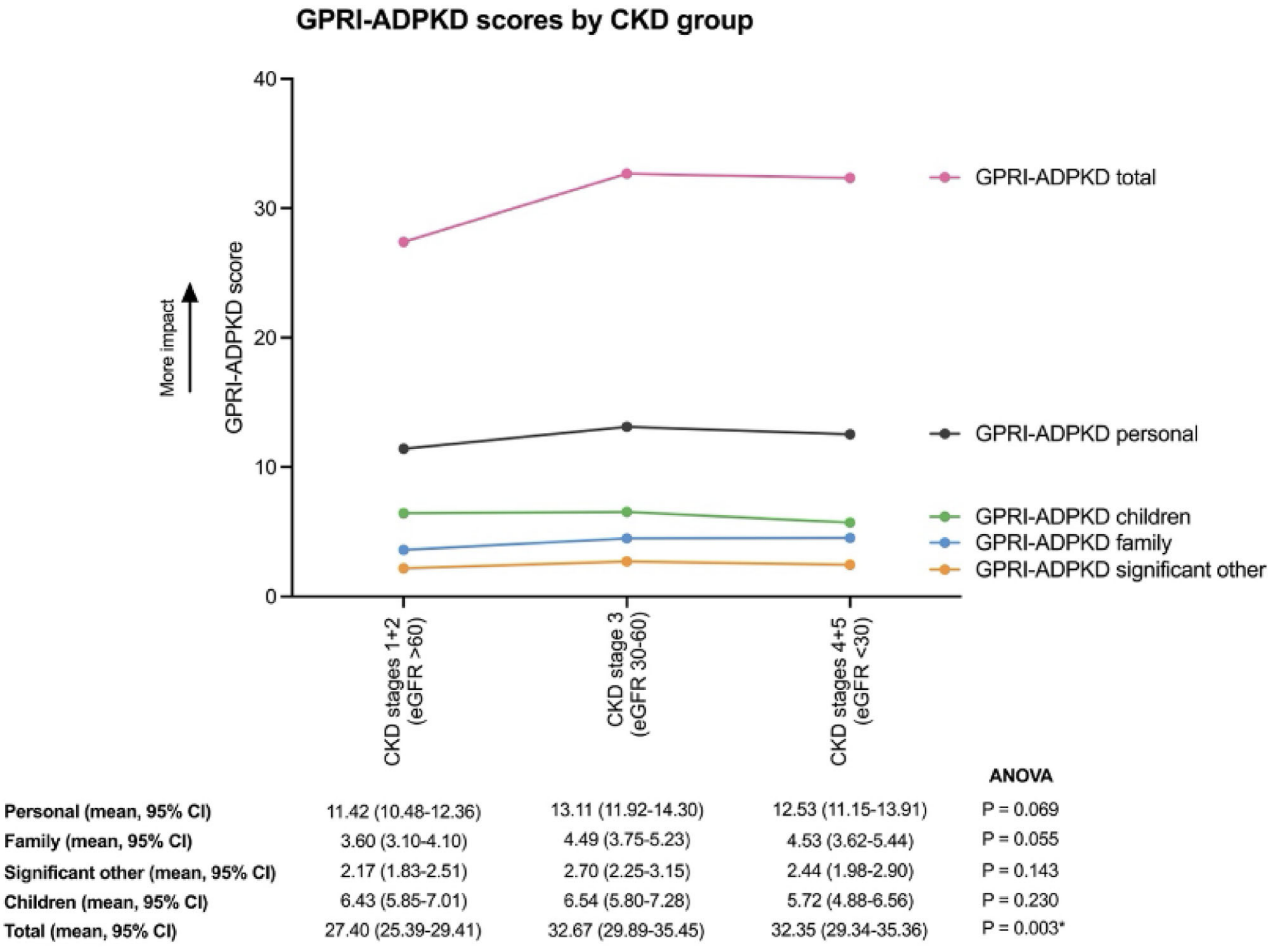
Mean GPRI-ADPKD scores by CKD stage.

Simms *et al*. reported GPRI-ADPKD scores that were developed to capture the impact of having a diagnosis of ADPKD on personal psychosocial risk and interpersonal relationships. The impact of an ADPKD diagnosis on quality of life was worse from CKD stages 1 + 2 compared to CKD stage 3 and 4 + 5, Figure 6 [27].

### Kidney volume and patient-reported outcomes

Five studies examined the relationship between kidney volume, measured as height-adjusted total kidney volume (htTKV) and kidney length (KL), and PROs in ADPKD (Table 4).

**Table 4:** Association between kidney size and PROs in ADPKD: summary of included studies.

Study author (year) (ref.)	Imaging	PROM	Statistical method	Main finding	Direction of association
Miskulin *et al*. (2014) [31]	htTKV	SF-36	Spearman rank (*ρ*)	No significant association with PCS or MCS	–
Perrone *et al*. (2023) [28]	htTKV	SF-12	Multivariable regression (*β*)	Significant association SF-12 with PCS (<.001) and MCS (.007)	Higher kidney size → worse PROs
Perrone *et al*. (2023) [28]	htTKV	ADPKD-IS	Multivariable regression (*β*)	Significant association with physical, emotional and fatigue scales	Higher kidney size → worse PROs
Simms *et al*. (2016) [27]	KL	SF-36	Group comparison (ANOVA)	PCS was significantly lower in KL ≥ 17 cm group; MCS showed no significant difference	Higher kidney size → worse physical PROs
Suwabe *et al*. (2017) [30]	htTKV	SF-36	Multivariable regression (*β*	No significant association with PCS or MCS	–
Winterbottom *et al*. (2022) [29]	htTKV	SF-36	Group comparison (ANOVA)	No significant association with PCS or MCS	–

Miskulin *et al*. and Suwabe *et al*. found no significant associations between htTKV and SF-36 PCS or MCS. Similarly Winterbottom *et al*. reported no significant differences in SF-36 scores across htTKV categories [29–31].

In contrast, Simms *et al*. observed that patients with larger kidneys (KL ≥ 17 cm) had significantly lower PCS scores, although MCS did not differ between groups [27]. Perrone *et al*. reported consistent findings across multiple instruments: higher htTKV was associate with worse SF-12 PCS and MCS scores, as well as worse disease-specific outcomes measured by the ADPKD-IS (physical, emotional, and fatigue domains) [28].

### Potential confounders

#### Age

Two studies acknowledged age as a potential influence on HRQoL [19, 27], but neither adjusted PROs to this factor. Simms *et al*. reported that increasing age was independently associated with lower PCS (*β* = −0.855, *P* < .001) and lower MCS (*β* = −4.280, *P* = .002) scores [27]. Eriksson *et al*. noted age differences between dialysis and transplant groups and the potential impact of age on extra-renal cyst prevalence but did not adjust PROs or assess age as an independent risk factor [19].

#### Sex

Most studies had a slightly higher proportion of female participants, but only three acknowledged the influence of sex on PROs [27, 29, 31], and none accounted for sex as a confounding factor between CKD group and PROs. Simms *et al*. reported that female sex was independently associated with lower PCS (*β* = −15.978, *P* = .001) and lower MCS (*β* = −18.305, *P* < .001) scores. Together, female sex and age explained 39% of the variance in PCS scores and 25% of variance in MCS scores [27].

Winterbottom *et al*. reported that female sex was independently associated with worse sleep (*β* = 6.018, 95% CI 1.973–10.063, *P* = .004) and greater pain (*β* = 6.348, 95% CI 1.941–2.241, *P* = .005) as measured by the SF-36 as part of the KDQoL PROM. Both effects remained significant after Bonferroni correction (*P* = .020 and .040, respectively). Female sex was also associated with higher symptom/problem burden, lower physical functioning, reduced emotional well-being, and lower energy/vitality scores, although these associations did not remain statistically significant after adjustment for multiple comparisons [29].

Miskulin *et al*. found males had better mean physical health (PCS 52.29 vs 50.38) and mental health (MCS 52.11 v 50.68), but did not adjust PROs for sex. Females reported higher bodily pain scores than males (mean 82.98 vs. 73.93). In particular, the severity of radicular pain derived from damage or irritation of spinal nerve tissue was greater in females (female 4.0 vs median male 3.0) [31]. In addition, females experienced more severe abdominal distension symptoms, including abdominal enlargement and reduced food intake, particularly at lower CKD stages (*P* < .05) [31].

#### Comorbidities

The potential impact of ADPKD or unrelated comorbidities between groups was acknowledged by three studies [19, 27, 28] but was not accounted for by adjusting PROs in any study. Simms *et al*. went further than other studies by demonstrating through multiple regression that comorbidity was independently associated with the PCS of SF-36 scores but the nature of these comorbidities or their association with CKD stage was not explored [27].

## DISCUSSION

### Main findings

This review synthesizes PROs for people with ADPKD using generic, kidney disease-specific, and ADPKD-specific instruments by CKD stage and KRT modality. As hypothesized, HRQoL worsened with advancing CKD stages and by KRT modality. Physical health and mental health declined with progressive CKD stages but mental health showed smaller deviations from relevant population norms. ADPKD transplant recipients had comparable physical health to people with ADPKD in late-stage CKD (stages 4 + 5) but this was lower than early-stage CKD (stages 1–3). However, mental health was not meaningfully different between people with ADPKD who were transplanted or had late-stage CKD.

The decline in physical health potentially reflects the progression of ADPKD from largely asymptomatic early stages to advanced stages with increased cyst burden and kidney enlargement that contribute to physical symptoms [19, 33]. Mental health showed less marked decline possibly due to psychological adaptation and acceptance observed in studies with other chronic illnesses [34–37], even as kidney failure approaches.

Our finding that transplant recipients had comparable physical and mental health to those with late-stage CKD as measured by SF PROMs, which contrasts with the findings of Ryu *et al*., who reported better post-transplant physical and mental health for CKD from any cause compared to CKD stages 1–3 [38]. Furthermore, EQ-5D scores were similar between transplanted recipients and late-stage CKD, unlike in any cause CKD as reported by Krishnan *et al*. who reported better PROs for transplant recipients than CKD stages 3–5 [39]. This discrepancy may arise because most people with ADPKD retain their polycystic kidneys post-transplant, so continue to experience physical symptoms related to kidney volume and cysts [31, 40, 41]. Transplanted ADPKD patients have persistent mental health issues related to genetic guilt, concerns about physical appearance, and unpredictability of symptoms, which are not dependent on CKD stage or KRT modality [42, 43].

An important consideration is that generic PROMs may not fully reflect the lived experiences of people with ADPKD, but could highlight limitations of these PROMs. Generic PROMs may lack construct validity, omitting ADPKD-specific patient-reported symptoms such as fatigue, abdominal fullness, urinary issues, and genetic guilt [40], potentially underestimating the burden of disease and influencing reimbursement decisions for therapies. Other rare and non-rare conditions have found similar findings [44]. This may be true for KDQoL-SF1.3 as the target scales did not decline as would be expected for people with ADPKD, this may be because KDQoL-SF1.3 focuses on end-stage kidney symptoms so may not capture changes in physical and mental health for people with early ADPKD. [27, 29]. By contrast, ADPKD-specific PROMs (ADPKD-IS, ADPKD-UIS, and GPRI-ADPKD) showed greater variation across CKD stages [27, 28] so may better represent the change in PROs across CKD stages in ADPKD.

### Strengths and limitations

This review synthesized available PROMs data for ADPKD across CKD stages and KRT modalities. Several limitations should be noted. Heterogeneity in study design, unequal participant numbers across CKD stages, and inconsistent use of PROMs made meta-analysis inappropriate. As PROMs rely on self-reported data, they are inherently susceptible to reporting biases. Multipliers were calculated relative to general population PROs; for some multinational studies lacking published reference populations, comparable populations were used that may have introduced approximation errors. Finally, in the primary studies confounders were not consistently evaluated or adjusted for age/sex-matched population norms.

### Clinical implications and future directions

Our findings underscore the importance of stage-specific, patient-centred management to describe the decline of PROs in ADPKD. Early interventions should target nocturia, fatigue, and work-related challenges before significant physical deterioration occurs. As CKD progresses, priority should include proactive pain management, cardiovascular risk reduction, and anaemia treatment. ADPKD-specific PROMs are essential to capture disease-specific concerns such as genetic anxiety, illness perception, and liver cyst burden. Presenting PROs as population multipliers provides general population-referenced estimates of quality of life decrement across CKD stages in ADPKD. These multipliers may help inform health economic modelling by informing assumptions about quality of life change across disease stages.

Several gaps remain. No quantitative evidence was identified for how CKD stage influences PROs for first-degree relatives at risk of inheriting ADPKDL. Standardized patient grouping is needed for future research to enable more direct comparisons between studies, with dialysis patients being categorized by modality and transplant patients categorized by CKD stage. Future studies should adjust confounders such as age and sex. Longitudinal research is needed to track changes in PROs over time, as current studies are cross-sectional.

### Conclusions

This review demonstrates that PROs for individuals with ADPKD are lower in later CKD stages compared to earlier stages, with the largest effect on physical health. Mental health scores are less affected which may suggest adaptation over time. Our findings suggest that generic PROMs may underestimate the impact of ADPKD compared to disease-specific tools.

## Supplementary Material

sfag116_Supplemental_File

## Data Availability

The datasets used and analysed in the current study are readily available from the authors of the primary studies.
