# Supplementary material for: Health-related quality of life in people with autosomal dominant polycystic kidney disease: a systematic review
Source: Clin Kidney J. 2026 Apr 23;19(6):sfag116. doi: 10.1093/ckj/sfag116 (PMC13223405; doi:10.1093/ckj/sfag116)
Supplement: sfag116_Supplemental_File [file sfag116_supplemental_file.docx]

# Appendices

### Appendix 1 – PECO framework and inclusion criteria

| PECO component | Description | Search terms and connections |
| --- | --- | --- |
| Population (P) | People with ADPKD | (ADPKD OR “Autosomal Dominant Polycystic Kidney” OR (Dominant AND (PKD OR ((Renal OR Kidney) N1 (cyst* OR multicystic OR cystic OR Polycystic))))) |
| Exposure (E) | HRQoL measurement tools (generic, kidney-specific or ADPKD-specific) | ("life quality" OR "quality of life" OR HRQOL OR QOL OR "mental health" OR ((health OR function* OR mental OR symptom*) N1 (status OR assess*)) OR "self report*" OR "patient* report*" OR "PROMs" OR "SF-36" OR "WHOQOL-BREF" OR "15D" OR "EQ-5D" OR "EQ5D" OR "KDQOL-SF" OR "ADPKD-IS" OR "ADPKD-impact scale" OR "PKD-9 questionnaire" OR "SF12" OR "SF-6D" OR "HUI" OR "VAS" OR "EuroQol" OR "EuroQual") |
| Comparator (C) | Comparison between different CKD stages | (ADPKD OR “Autosomal Dominant Polycystic Kidney” OR (Dominant AND (PKD OR ((Renal OR Kidney) N1 (cyst* OR multicystic OR cystic OR Polycystic))))) |
| Outcome (O) | HRQoL outcomes | ("life quality" OR "quality of life" OR HRQOL OR QOL OR "mental health" OR ((health OR function* OR mental OR symptom*) N1 (status OR assess*)) OR "self report*" OR "patient* report*" OR "PROMs" OR "SF-36" OR "WHOQOL-BREF" OR "15D" OR "EQ-5D" OR "EQ5D" OR "KDQOL-SF" OR "ADPKD-IS" OR "ADPKD-impact scale" OR "PKD-9 questionnaire" OR "SF12" OR "SF-6D" OR "HUI" OR "VAS" OR "EuroQol" OR "EuroQual") |

### Appendix 2 – Search strategies

**Medline OVID:**

1. (ADPKD or "Autosomal Dominant Polycystic Kidney" or (Dominant and (PKD or ((Renal or Kidney) adj1 (cyst* or failure* or multicystic or cystic or Polycystic))))).mp.

("life quality" or "quality of life" or HRQOL or QOL or "mental health" or ((health or function* or mental or symptom*) adj1 (status or assess*)) or "self report*" or "patient* report*" or "PROMs" OR "SF-36" OR "WHOQOL-BREF" OR "15D" OR "EQ-5D" OR "EQ5D" OR "KDQOL-SF" OR "ADPKD-IS" OR "ADPKD-impact scale" OR "PKD-9 questionnaire" OR "SF12" OR "SF-6D" OR "HUI" OR "VAS" OR "EuroQol" OR "EuroQual").mp.

3 1 and 2

4 3 not ("Qualitative Studies" or "Case Reports" or "Editorials" or "Commentaries" or "Conference Abstracts" or "Protocols").ti.

5 limit 4 to (english language and yr="2014 -Current" and (adaptive clinical trial or classical article or clinical study or clinical trial, all or clinical trial, phase i or clinical trial, phase ii or clinical trial, phase iii or clinical trial, phase iv or clinical trial or comparative study or controlled clinical trial or "corrected and republished article" or evaluation study or journal article or meta analysis or multicenter study or observational study or pragmatic clinical trial or randomized controlled trial or "systematic review") and "humans only (removes records about animals)")

**Psyclnfo OVID:**

1.(ADPKD or "Autosomal Dominant Polycystic Kidney" or (Dominant and (PKD or ((Renal or Kidney) adj1 (cyst* or multicystic or cystic or Polycystic))))).mp.

2 ("life quality" or "quality of life" or HRQOL or QOL or "mental health" or ((health or function* or mental or symptom*) adj1 (status or assess*)) or "self report*" or "patient* report*" or "PROMs" OR "SF-36" OR "WHOQOL-BREF" OR "15D" OR "EQ-5D" OR "EQ5D" OR "KDQOL-SF" OR "ADPKD-IS" OR "ADPKD-impact scale" OR "PKD-9 questionnaire" OR "SF12" OR "SF-6D" OR "HUI" OR "VAS" OR "EuroQol" OR "EuroQual").mp.

3. 1 and 2

4. 3 not ("Qualitative Studies" or "Case Reports" or "Editorials" or "Commentaries" or "Conference Abstracts" or "Protocols").ti.

5. limit 4 to (all journals and human and english language and "0110 peer-reviewed journal" and yr="2014 -Current")

**Embase OVID:**

1 (ADPKD or "Autosomal Dominant Polycystic Kidney" or (Dominant and (PKD or ((Renal or Kidney) adj1 (cyst* or multicystic or cystic or Polycystic))))).mp.

2 ("life quality" or "quality of life" or HRQOL or QOL or "mental health" or ((health or function* or mental or symptom*) adj1 (status or assess*)) or "self report*" or "patient* report*" or "PROMs" OR "SF-36" OR "WHOQOL-BREF" OR "15D" OR "EQ-5D" OR "EQ5D" OR "KDQOL-SF" OR "ADPKD-IS" OR "ADPKD-impact scale" OR "PKD-9 questionnaire" OR "SF12" OR "SF-6D" OR "HUI" OR "VAS" OR "EuroQol" OR "EuroQual").mp.

3 1 and 2

4 3 not ("Qualitative Studies" or "Case Reports" or "Editorials" or "Commentaries" or "Conference Abstracts" or "Protocols").ti.

5 limit 4 to (human and english language and "remove medline records" and (article or article in press) and yr="2014 -Current")

**CINAHL via EBSCO:**

( ADPKD OR “Autosomal Dominant Polycystic Kidney” OR (Dominant AND (PKD OR ((Renal OR Kidney) N1 (cyst* OR multicystic OR cystic OR Polycystic)))) )

AND

("life quality" OR "quality of life" OR HRQOL OR QOL OR "mental health" OR ((health OR function* OR mental OR symptom*) N1 (status OR assess*)) OR "self report*" OR "patient* report*" OR "PROMs" OR "SF-36" OR "WHOQOL-BREF" OR "15D" OR "EQ-5D" OR "EQ5D" OR "KDQOL-SF" OR "ADPKD-IS" OR "ADPKD-impact scale" OR "PKD-9 questionnaire" OR "SF12" OR "SF-6D" OR "HUI" OR "VAS" OR "EuroQol" OR "EuroQual")

NOT

TI ( ("Qualitative Studies" OR "Case Reports" OR "Editorials" OR "Commentaries" OR "Conference Abstracts" OR "Protocols") )

Limiters - Publication Date: 20140101-20241231; English Language; Peer Reviewed; Exclude Pre-CINAHL; Exclude MEDLINE records; Human

Expanders - Apply equivalent subjects

Search modes - Proximity

**Web of Science:**

TS=(ADPKD OR "Autosomal Dominant Polycystic Kidney" OR (Dominant AND (PKD OR ((Renal OR Kidney) NEAR/1 (cyst* OR multicyclic OR cystic OR Polycystic))))) AND TS=("life quality" OR "quality of life" OR HRQOL OR QOL OR "mental health" OR ((health OR function* OR mental OR symptom*) NEAR/1 (status OR assess*)) OR "self report*" OR "patient* report*" OR "PROMs" OR "SF-36" OR "WHOQOL-BREF" OR "15D" OR "EQ-5D" OR "EQ5D" OR "KDQOL-SF" OR "ADPKD-IS" OR "ADPKD-impact scale" OR "PKD-9 questionnaire" OR "SF12" OR "SF-6D" OR "HUI" OR "VAS" OR "EuroQol" OR "EuroQual")

AND LA=(English) AND PY=(2014-2024) and Preprint Citation Index (Exclude – Database) and MEDLINE® (Exclude – Database) and Article or Review Article or Early Access (Document Types) and Book or Meeting or Editorial Material (Exclude – Document Types)

**Google Scholar:**

Search "ADPKD HRQOL" and retrieve the first 30 hits.

### Appendix 3 – Study characteristics (detailed)

|  |  |  |  |  |  |  | Population characteristics | | | | | | | |
| --- | --- | --- | --- | --- | --- | --- | --- | --- | --- | --- | --- | --- | --- | --- |
| Study author (year) | Country of 1^st^ author | Participating countries (n) | Funding source | Study design | Design type | PROMS (n) | Participants (n) | ADPKD diagnosis | CKD stages | Age range | Mean age (SD) | Gender (% female) | Ethnicity (% White) | ADPKD genotype |
| Eriksson et al. (2017) | Sweden | United Kingdom, Denmark, Sweden (3) | Pharmaceutical companies (Otsuka) | Cross-sectional | Self-administered questionnaire | EQ-5D, SF-12, WPAI:GH (3) | 243 | NR | CKD 1-5, 5D, 5T | 18 years or more | 58.0 (12.1) | 53.9 | NR | NR |
| Miskulin et al. (2014) | United States | United States (1) | Research organisations (National Institute for Diabetes and Digestive and Kidney Diseases, NIH, National Centre for Research Resources General Clinical Research Centers, Centers for Translational Science Activities) | Cross-sectional | Self-administered questionnaire | SF-36, WBPS (2) | 1043 | NR | CKD1-4 | 15-64 years | 41.7 (10.3) | 49.9 | 93.0 | NR |
| Perrone  et al. (2023) | United States | Argentina, Australia, Belgium, Brazil, Canada, Czech Republic, France, Germany, Italy, Japan, Netherlands, Norway, Poland, Romania, Spain, Sweden, Switzerland, Turkey, United Kingdom, United States (20) | Pharmaceutical company (Otsuka) | Longitudinal | Self-administered questionnaire | BPI-SF, CPS, EQ-5D, SF-12, ADPKD-IS, ADPKD-UIS (6) | 3409 | Pei-Ravine 2009 | CKD 1-5, 5D | 12-70 years | 45.1 (12.9) | 55.5 | 85.6 | NR |
| Simms et al. (2016) | United Kingdom | United Kingdom (1) | NHS trust research foundation (Sheffield Kidney Research Foundation) | Cross-sectional | Unclear | MSPSS, SF-36, KDQoL-SF, GPRI-ADPKD (3) | 349 | Ravine 1994 | CKD 1-5 | 18 years or more | 53.4 (15.8) | 58.3 | 95.0 | NR |
| Suwabe et al. (2017) | Japan | Japan (1) | Research organisations and hospitals (Japanese Association of Dialysis, Kidney Foundation, Ministry of Health, Okinaka Memorial Institute for Medical Research, Toranomon Hospital) | Longitudinal | Self-administered | SF-36 (1) | 188 | Pei-Ravine 2009 & Progressive renal disease research from the ministry of health, labour and welfare of Japan | CKD 5T | 20 year or more | 56.7 (9.1) | 51.1 | NR | NR |
| Winterbottom et al. (2022) | United Kingdom | Belgium, France, Italy, Netherlands, Spain, United Kingdom (6) | Multiple sources (Otsuka, Sheffield Kidney Research Foundation, PKD Charity) | Cross-sectional | Unclear | KDQoL-SF (1) | 465 | Pei-Ravine 2009 | CKD 1-3 | 18 years or more | 43.2 (12.8) | 55.1 | 96.3 | PKD1, PKD2 |

### Appendix 4 –

| **PROMs** | **CKD stage** | **N studies** | **I2 (%)** |
| --- | --- | --- | --- |
| SF-12/36: PCS | Overall | 6 | 99.6 |
|  | Early | 5 | 99.8 |
|  | Late | 4 | 98.0 |
|  | Dialysis | 1 | 96.3 |
|  | Transplant | 2 | - |
| SF-12/36: MCS | Overall | 6 | 99.2 |
|  | Early | 5 | 99.4 |
|  | Late | 4 | 97.5 |
|  | Dialysis | 1 | 99.3 |
|  | Transplant | 2 | - |
|  | Overall | 4 | 95.8 |
|  | Early | 4 | 93.4 |
|  | Late | 4 | 10.4 |
|  | Dialysis | 3 | 7.4 |
|  | Transplant | 3 | 56.1 |

### Appendix 5 – Raw PROMs scores unadjusted to reference general population


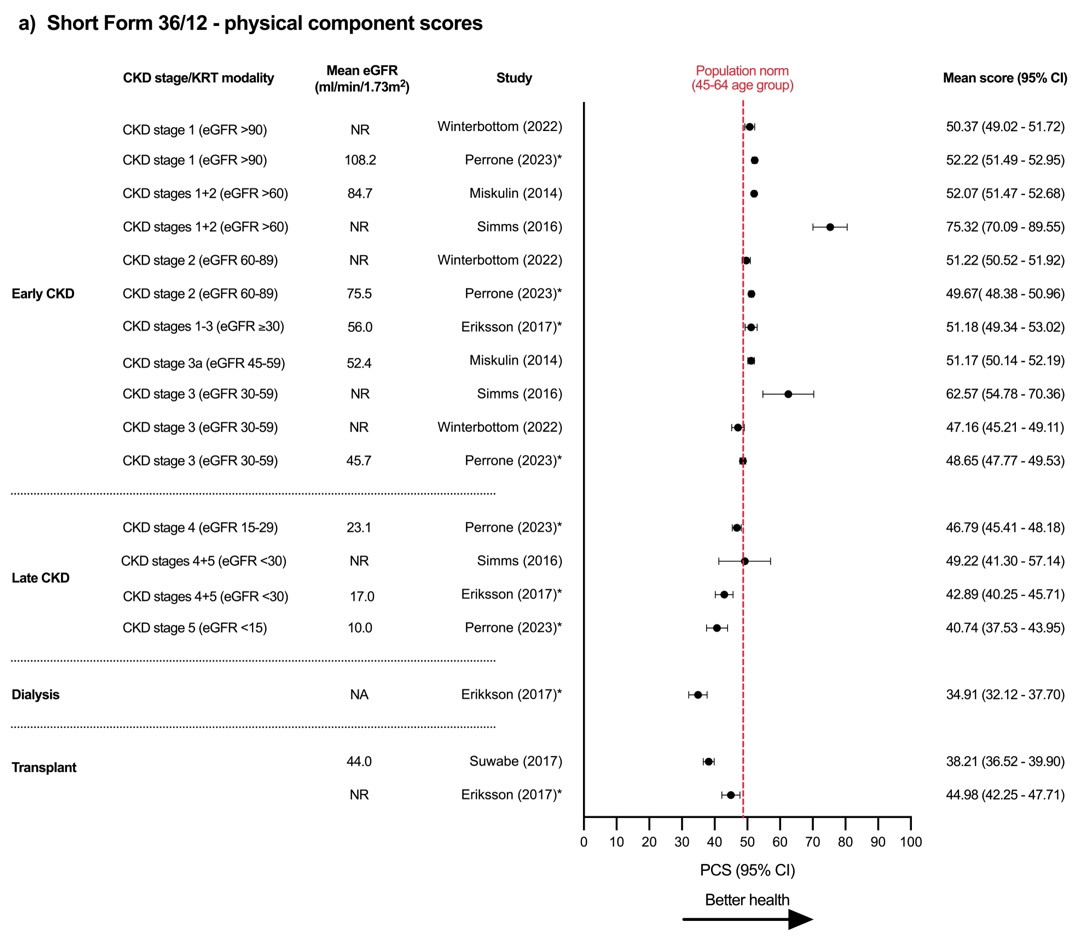


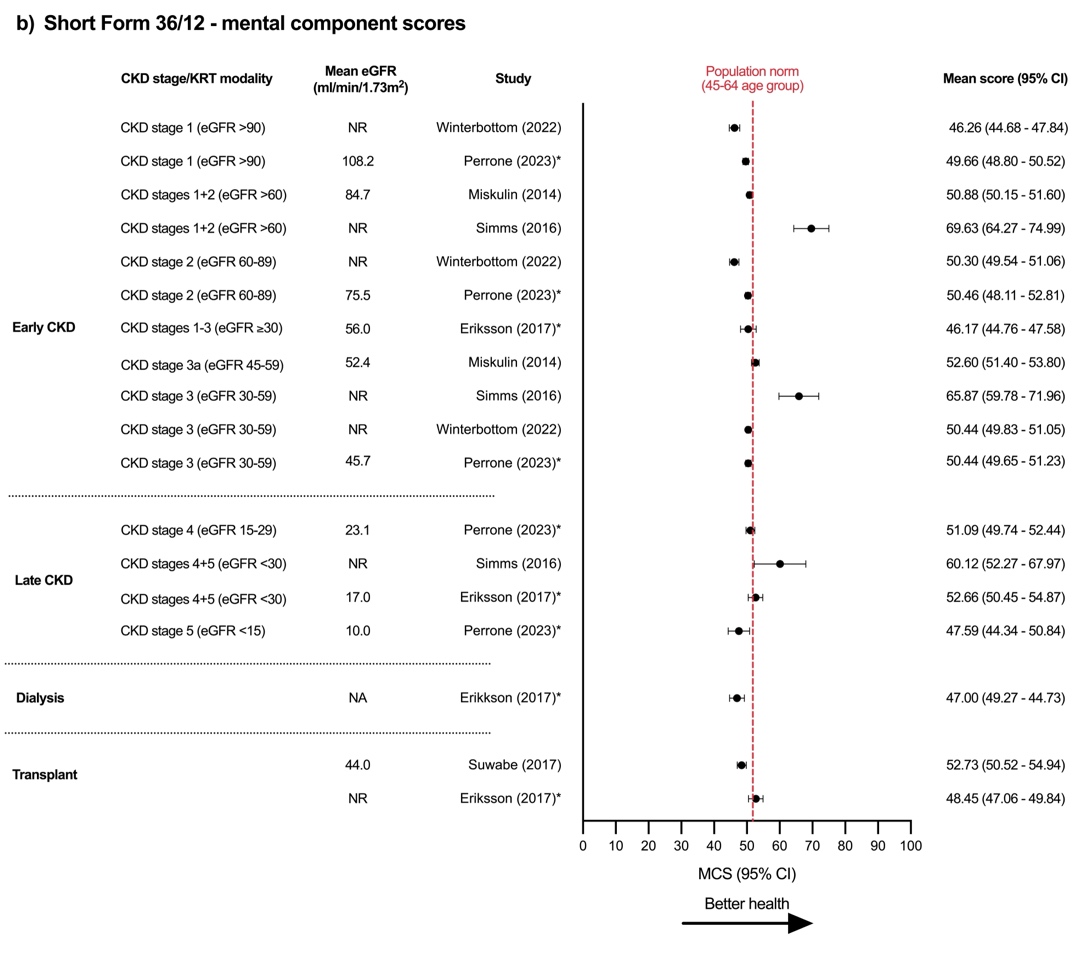


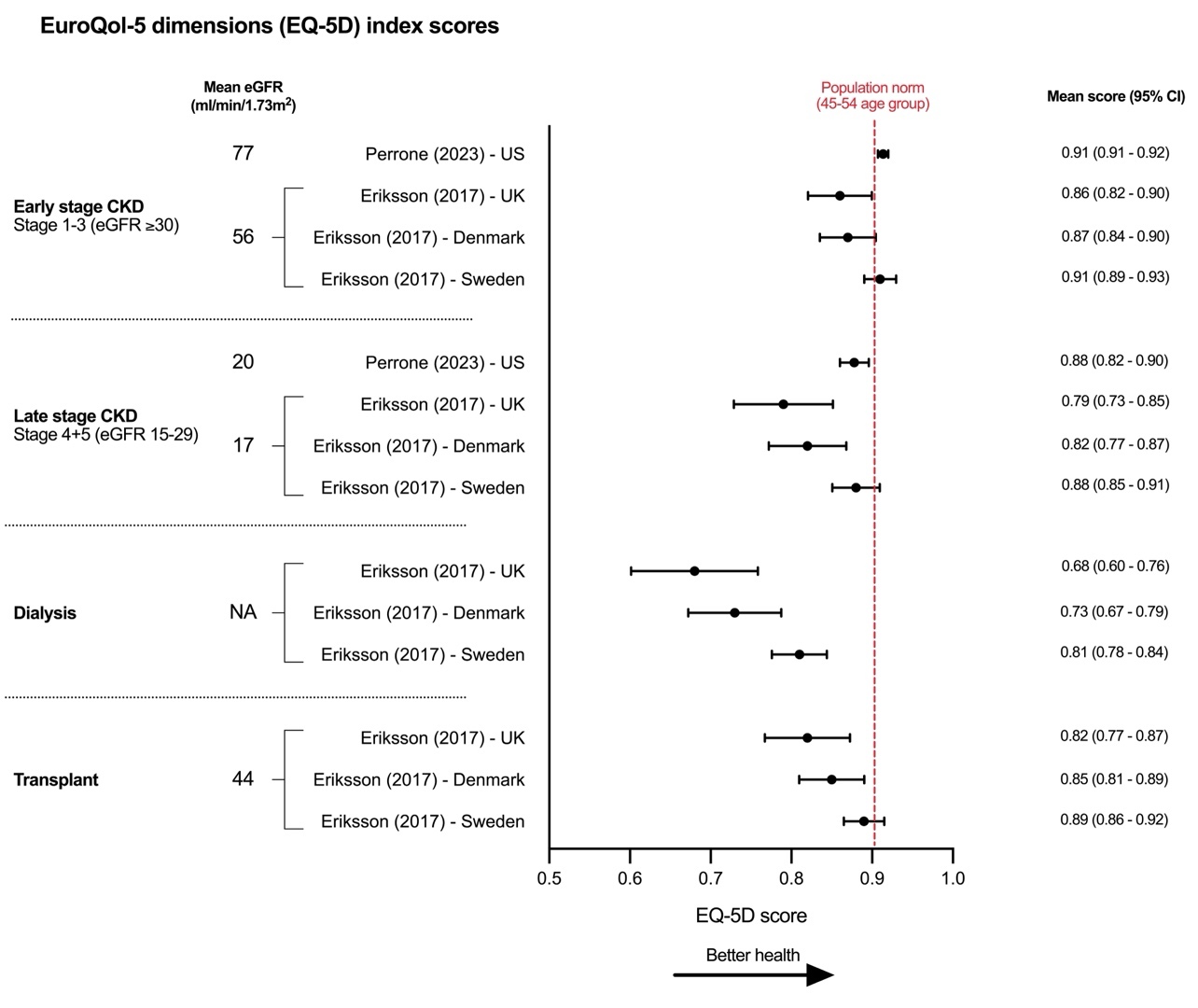


### Appendix 6 – SF-12/36 dimensions across CKD stage/KRT modality


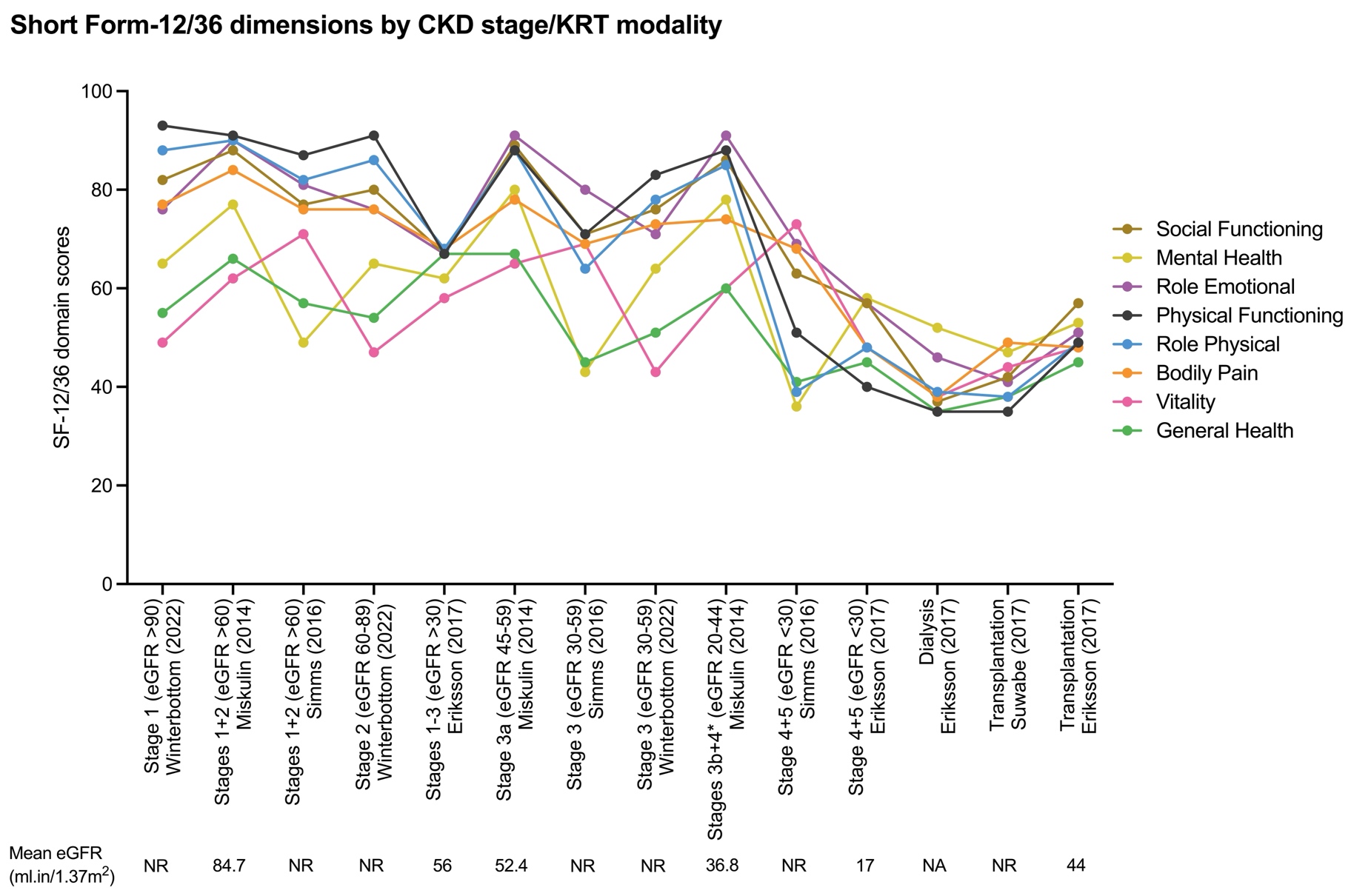


### Appendix 7 – Reference populations for patient reported outcome measures used for studies

| Author (year) | Subgroup | Country | Mean age | Reference general population | Country | Age group |
| --- | --- | --- | --- | --- | --- | --- |
| Eriksson et al. (2017) | Stages 1-3 | Denmark, Sweden, UK | 52 | Jenkinson et al. (1993). Short form 36 (SF36) health survey questionnaire: normative data for adults of working age | UK | 45-54 |
| Eriksson et al. (2017) | Stages 4-5 | Denmark, Sweden, UK | 57 | Jenkinson et al. (1993). Short form 36 (SF36) health survey questionnaire: normative data for adults of working age | UK | 55-64 |
| Eriksson et al. (2017) | Dialysis | Denmark, Sweden, UK | 64 | Jenkinson et al. (1993). Short form 36 (SF36) health survey questionnaire: normative data for adults of working age | UK | 55-64 |
| Eriksson et al. (2017) | Transplant | Denmark, Sweden, UK | 59 | Jenkinson et al. (1993). Short form 36 (SF36) health survey questionnaire: normative data for adults of working age | UK | 55-64 |
| Winterbottom et al. (2022) | Stage 1 | Europe | 33 | Jenkinson et al. (1993). Short form 36 (SF36) health survey questionnaire: normative data for adults of working age | UK | 25-34 |
| Winterbottom et al. (2022) | Stage 2 | Europe | 44 | Jenkinson et al. (1993). Short form 36 (SF36) health survey questionnaire: normative data for adults of working age | UK | 35-44 |
| Winterbottom et al. (2022) | Stage 3 | Europe | 53 | Jenkinson et al. (1993). Short form 36 (SF36) health survey questionnaire: normative data for adults of working age | UK | 45-54 |
| Winterbottom et al. (2022) | Stage 4 | Europe | 57 | Jenkinson et al. (1993). Short form 36 (SF36) health survey questionnaire: normative data for adults of working age | UK | 55-64 |
| Simms et al.  (2016) | Stages 1-2 | UK | 45 | Jenkinson et al. (1993). Short form 36 (SF36) health survey questionnaire: normative data for adults of working age | UK | 45-54 |
| Simms et al.  (2016) | Stage 3 | UK | 56 | Jenkinson et al. (1993). Short form 36 (SF36) health survey questionnaire: normative data for adults of working age | UK | 55-64 |
| Simms et al.  (2016) | Stages 4-5 | UK | 66 | Jenkinson et al. (1993). Short form 36 (SF36) health survey questionnaire: normative data for adults of working age | UK | 55-64 |
| Miskulin et al. (2014) | Stages 1-2 | US | 37 | Maglinte et al. (2013). US general population norms for telephone administration of the SF-36v2 | US | 35-44 |
| Miskulin et al. (2014) | Stage 3a | US | 47 | Maglinte et al. (2013). US general population norms for telephone administration of the SF-36v2 | US | 45-54 |
| Miskulin et al. (2014) | Stages 3+4 | US | 49 | Maglinte et al. (2013). US general population norms for telephone administration of the SF-36v2 | US | 45-54 |
| Suwabe et al. (2017) | Transplant | Japan | 57 | Mitoma et al. (2016). Prevalence of chronic pain, especially headache, and relationship with health-related quality of life in middle-aged Japanese residents | Japan | 50-59 |
| Perrone et al. (2023) | Stage 1 | US | 34 | Maglinte et al. (2013). US general population norms for telephone administration of the SF-36v2 | US | 35-44 |
| Perrone et al. (2023) | Stage 2 | US | 45 | Maglinte et al. (2013). US general population norms for telephone administration of the SF-36v2 | US | 45-54 |
| Perrone et al. (2023) | Stage 3 | US | 52 | Maglinte et al. (2013). US general population norms for telephone administration of the SF-36v2 | US | 45-54 |
| Perrone et al. (2023) | Stage 4 | US | 53 | Maglinte et al. (2013). US general population norms for telephone administration of the SF-36v2 | US | 45-54 |
| Perrone et al. (2023) | Stage 5 | US | 55 | Maglinte et al. (2013). US general population norms for telephone administration of the SF-36v2 | US | 55-64 |

# Supplementary Materials

### Supplementary table 1 – PRISMA 2020 statement

| **Section and Topic** | **Item #** | **Checklist item** | **Location where item is reported** |
| --- | --- | --- | --- |
| **TITLE** | | |  |
| Title | 1 | Identify the report as a systematic review. | Page 1 |
| **ABSTRACT** | | |  |
| Abstract | 2 | See the PRISMA 2020 for Abstracts checklist. | Page 2 |
| **INTRODUCTION** | | |  |
| Rationale | 3 | Describe the rationale for the review in the context of existing knowledge. | Page 5 |
| Objectives | 4 | Provide an explicit statement of the objective(s) or question(s) the review addresses. | Page 5 |
| **METHODS** | | |  |
| Eligibility criteria | 5 | Specify the inclusion and exclusion criteria for the review and how studies were grouped for the syntheses. | Page 6 |
| Information sources | 6 | Specify all databases, registers, websites, organisations, reference lists and other sources searched or consulted to identify studies. Specify the date when each source was last searched or consulted. | Page 6 |
| Search strategy | 7 | Present the full search strategies for all databases, registers and websites, including any filters and limits used. | Appendix 2 |
| Selection process | 8 | Specify the methods used to decide whether a study met the inclusion criteria of the review, including how many reviewers screened each record and each report retrieved, whether they worked independently, and if applicable, details of automation tools used in the process. | Page 6 |
| Data collection process | 9 | Specify the methods used to collect data from reports, including how many reviewers collected data from each report, whether they worked independently, any processes for obtaining or confirming data from study investigators, and if applicable, details of automation tools used in the process. | Page 7 |
| Data items | 10a | List and define all outcomes for which data were sought. Specify whether all results that were compatible with each outcome domain in each study were sought (e.g. for all measures, time points, analyses), and if not, the methods used to decide which results to collect. | Page 7 |
|  | 10b | List and define all other variables for which data were sought (e.g. participant and intervention characteristics, funding sources). Describe any assumptions made about any missing or unclear information. | Page 7 |
| Study risk of bias assessment | 11 | Specify the methods used to assess risk of bias in the included studies, including details of the tool(s) used, how many reviewers assessed each study and whether they worked independently, and if applicable, details of automation tools used in the process. | Page 7 |
| Effect measures | 12 | Specify for each outcome the effect measure(s) (e.g. risk ratio, mean difference) used in the synthesis or presentation of results. | Page 7 |
| Synthesis methods | 13a | Describe the processes used to decide which studies were eligible for each synthesis (e.g. tabulating the study intervention characteristics and comparing against the planned groups for each synthesis (item #5)). | Page 7 |
|  | 13b | Describe any methods required to prepare the data for presentation or synthesis, such as handling of missing summary statistics, or data conversions. | Page 7 |
|  | 13c | Describe any methods used to tabulate or visually display results of individual studies and syntheses. | Page 7 |
|  | 13d | Describe any methods used to synthesize results and provide a rationale for the choice(s). If meta-analysis was performed, describe the model(s), method(s) to identify the presence and extent of statistical heterogeneity, and software package(s) used. | Page 7 |
|  | 13e | Describe any methods used to explore possible causes of heterogeneity among study results (e.g. subgroup analysis, meta-regression). | Page 7 |
|  | 13f | Describe any sensitivity analyses conducted to assess robustness of the synthesized results. | Page 7 |
| Reporting bias assessment | 14 | Describe any methods used to assess risk of bias due to missing results in a synthesis (arising from reporting biases). | Page 7 |
| Certainty assessment | 15 | Describe any methods used to assess certainty (or confidence) in the body of evidence for an outcome. | NA |
| **RESULTS** | | |  |
| Study selection | 16a | Describe the results of the search and selection process, from the number of records identified in the search to the number of studies included in the review, ideally using a flow diagram. | Page 8 |
|  | 16b | Cite studies that might appear to meet the inclusion criteria, but which were excluded, and explain why they were excluded. | Page 8 |
| Study characteristics | 17 | Cite each included study and present its characteristics. | Page 9 |
| Risk of bias in studies | 18 | Present assessments of risk of bias for each included study. | Page 11 |
| Results of individual studies | 19 | For all outcomes, present, for each study: (a) summary statistics for each group (where appropriate) and (b) an effect estimate and its precision (e.g. confidence/credible interval), ideally using structured tables or plots. | Page 11-13 |
| Results of syntheses | 20a | For each synthesis, briefly summarise the characteristics and risk of bias among contributing studies. | Pages 9-11 |
|  | 20b | Present results of all statistical syntheses conducted. If meta-analysis was done, present for each the summary estimate and its precision (e.g. confidence/credible interval) and measures of statistical heterogeneity. If comparing groups, describe the direction of the effect. | Page 11 |
|  | 20c | Present results of all investigations of possible causes of heterogeneity among study results. | Page 11 |
|  | 20d | Present results of all sensitivity analyses conducted to assess the robustness of the synthesized results. | NA |
| Reporting biases | 21 | Present assessments of risk of bias due to missing results (arising from reporting biases) for each synthesis assessed. | NA |
| Certainty of evidence | 22 | Present assessments of certainty (or confidence) in the body of evidence for each outcome assessed. | NA |
| **DISCUSSION** | | |  |
| Discussion | 23a | Provide a general interpretation of the results in the context of other evidence. | Page 17-18 |
|  | 23b | Discuss any limitations of the evidence included in the review. | Page 18 |
|  | 23c | Discuss any limitations of the review processes used. | Page 18 |
|  | 23d | Discuss implications of the results for practice, policy, and future research. | Page 18-19 |
| **OTHER INFORMATION** | | |  |
| Registration and protocol | 24a | Provide registration information for the review, including register name and registration number, or state that the review was not registered. | Page 5 |
|  | 24b | Indicate where the review protocol can be accessed, or state that a protocol was not prepared. | Page 5 |
|  | 24c | Describe and explain any amendments to information provided at registration or in the protocol. | NA |
| Support | 25 | Describe sources of financial or non-financial support for the review, and the role of the funders or sponsors in the review. | Page 20 |
| Competing interests | 26 | Declare any competing interests of review authors. | Page 19 |
| Availability of data, code and other materials | 27 | Report which of the following are publicly available and where they can be found: template data collection forms; data extracted from included studies; data used for all analyses; analytic code; any other materials used in the review. | Appendices |

*From:*  Page MJ, McKenzie JE, Bossuyt PM, Boutron I, Hoffmann TC, Mulrow CD, et al. The PRISMA 2020 statement: an updated guideline for reporting systematic reviews. BMJ 2021;372:n71. doi: 10.1136/bmj.n71. This work is licensed under CC BY 4.0. To view a copy of this license, visit <https://creativecommons.org/licenses/by/4.0/>

### Supplementary table 2 – Summary of patient reported outcome measures

| **Health Related Quality of Life Assessment Tools** | |
| --- | --- |
| Generic assessment tools | |
| EuroQol-5D (EQ-5D) | Generates a score based on five health domains typically from -0.594 (worst health) to 1 (perfect health), with scores below zero indicating health states considered worse than death. |
| Short Form-12 (SF-12) | Two summary scores for physical and mental health (0-100) from 8 domains, where higher scores represent better health. Shorter version than SF-36. |
| Short Form-36 (SF-36) | Two summary scores for physical and mental health (0-100) from 8 domains, where higher scores represent better health. |
| Kidney disease assessment tools | |
| Kidney Disease Quality of Life - Short Form (KDQoL-SF) | Includes SF-36 and additional kidney-specific items, with scores ranging from 0 to 100, where higher scores reflect better quality of life. |
| ADPKD-specific assessment tools | |
| Autosomal Dominant Polycystic Kidney Disease-Impact Score (ADPKD-IS) | Scored 0-100, with higher scores reflecting greater impact of the disease. |
| Autosomal Dominant Polycystic Kidney Disease-Urinary Impact Score (ADPKD-UIS) | Scored 0-100, with higher scores reflecting greater impact of urinary symptoms. |
| General Polycystic Kidney Disease-Related Impact (GPRI-ADPKD) | Scored 0-100, assessing overall impact of ADPKD on quality of life, with higher scores showing greater impact. |

### Supplementary table 3 – Justification of quality assessment

| Study author (year) | Q1. Eligibility criteria | Q2. Subjects and Setting | Q3. Exposure measurement | Q4. Measurement condition | Q5. Confounders | Q6. Strategies for confounders | G7. Validity of the Outcomes | G8. Statistical analysis |
| --- | --- | --- | --- | --- | --- | --- | --- | --- |
| Eriksson et al. (2017) [1] | Methods p. 2107 | Results p. 2108 | NA | Unclear | No. Age and comorbidities are mentioned in the discussion but age and sex are not considered in the referenced general population set | Not mentioned | EQ-5D-3L & SF-12v2 | Descriptive and norm based summary |
| Miskulin et al. (2014) [2] | In a previous publication | Table 1 and a previous publication | ACE inhibitor/ARB vs ACE-I, described in a previous publication | Unclear | Yes. Age and sex are considered in the referenced general population set | Not mentioned | SF-36; Wisconsin Brief Pain  Survey | Descriptive and norm based summary |
| Perrone et al. (2023) [3] | Methods p. 990 | Table 1 | NA | Ravine criteria | Age and cyst burden | Figure 1 and discussion | EQ-5D-3L & SF-12v2 | Descriptive and norm based summary |
| Simms et al. (2016) [4] | Methods p. 1131 | Table 1 | NA | Ravine criteria | Yes. Age and sex considered | Table 6 | MSPSS, SF-36, KDQoL-SF, GPRI-ADPKD | Descriptive and norm based summary |
| Suwabe et al. (2017) [5] | Methods table 1 | Table 2 | Transcatheter arterial embolisation | Pei criteria | No confounders mentioned. | Not mentioned | SF-36 | Descriptive and norm based summary |
| Winterbottom et al. (2022) [6] | Methods p. 2065 | Table 1 & 4 | NA | Ravine criteria | No. Sex only. | Supplementary table 5 | KDQoL-SF | Descriptive and norm based summary |

Q1. Were the criteria for inclusion in the sample clearly defined?

Q2 Were the study subjects and the setting described in detail?

Q3. Was the exposure measured in a valid and reliable way?

Q4. Were objective, standard criteria used for measurement of the condition?

Q5. Were confounding factors identified?

Q6. Were strategies to deal with confounding factors stated?

Q7. Were the outcomes measured in a valid and reliable way?

Q8. Was appropriate statistical analysis used?

### Supplementary table 4 – Individual domains of PROMs

### Unadjusted SF-36 and SF-12 scores

| **Study, year** | **CKD stage** | **PF** | **RP** | **BP** | **GH** | **V** | **SF** | **RE** | **MH** | **PCS** | **MCS** |
| --- | --- | --- | --- | --- | --- | --- | --- | --- | --- | --- | --- |
| Winterbottom, 2022 | Stage 1 | 92 | 89 | 77 | 55 | 50 | 92 | 76 | 65 | 50.20 | 46.34 |
| Perrone, 2023 | Stage 1 | - | - | - | - | - | - | - | - | 52.22 | 49.66 |
| Simms, 2016 | Stage 1+2 | 87 | 82 | 76 | 57 | 71 | 77 | 81 | 49 | 75.32 | 69,63 |
| Miskulin, 2014 | Stage 1+2 | 91 | 90 | 84 | 66 | 62 | 88 | 90 | 77 | 52.07 | 51.39 |
| Winterbottom, 2022 | Stage 2 | 91 | 86 | 76 | 55 | 48 | 91 | 77 | 66 | 49.72 | 46.62 |
| Perrone, 2023 | Stage 2 | - | - | - | - | - | - | - | - | 51.22 | 50.30 |
| Miskulin, 2014 | Stage 3a | 88 | 88 | 78 | 67 | 65 | 89 | 91 | 80 | 51.15 | 52.61 |
| Eriksson, 2017 | Stage 1-3 | 67 | 68 | 68 | 67 | 58 | 67 | 67 | 62 | 51.12 | 50.46 |
| Winterbottom, 2022 | Stage 3 | 83 | 78 | 73 | 51 | 44 | 83 | 71 | 64 | 47.29 | 45.67 |
| Perrone, 2023 | Stage 3 | - | - | - | - | - | - | - | - | 48.65 | 50.44 |
| Simms, 2016 | Stage 3 | 71 | 64 | 69 | 45 | 69 | 71 | 80 | 43 | 62.57 | 65.87 |
| Miskulin, 2014 | Stage 3b+4* | 88 | 85 | 74 | 60 | 60 | 86 | 91 | 78 | 49.37 | 51.56 |
| Winterbottom, 2022 | Stage 4 | 84 | 89 | 76 | 54 | 38 | 84 | 76 | 62 | 44.87 | 46.95 |
| Perrone, 2023 | Stage 4 | - | - | - | - | - | - | - | - | 46.79 | 51.09 |
| Eriksson, 2017 | Stage 4+5 | 40 | 48 | 48 | 45 | 48 | 57 | 57 | 58 | 42.98 | 52.66 |
| Simms, 2016 | Stage 4+5 | 51 | 39 | 68 | 41 | 73 | 63 | 69 | 36 | 49.22 | 60.12 |
| Perrone, 2023 | Stage 5 | - | - | - | - | - | - | - | - | 40.74 | 47.59 |
| Eriksson, 2017 | Dialysis | 35 | 39 | 38 | 35 | 38 | 37 | 46 | 52 | 34.91 | 47.00 |
| Suwabe, 2017 | Transplant | 35 | 38 | 49 | 38 | 44 | 42 | 41 | 47 | 38.21 | 48.45 |
| Eriksson, 2017 | Transplant | 49 | 49 | 48 | 45 | 48 | 57 | 51 | 53 | 44.98 | 52.73 |

** SF-12*

### EQ-5D-3L

| **Study, year** | **Country** | **CKD 1-3** | **CKD 4-5** | **Dialysis** | **Transplant** |
| --- | --- | --- | --- | --- | --- |
| Eriksson (2017) | Denmark | 0.87 | 0.82 | 0.73 | 0.85 |
| Eriksson (2017) | Sweden | 0.91 | 0.88 | 0.81 | 0.89 |
| Eriksson (2017) | United Kingdom | 0.86 | 0.79 | 0.68 | 0.82 |
| Perrone (2017) | Global | 0.91 | 0.88 | - | - |

*

*Total mean – not reported for individual countries*
